# (*E*)-4-*tert*-Butyl-2-(2,6-diisopropyl­phenyl­imino­meth­yl)-6-(morpholinometh­yl)phenol

**DOI:** 10.1107/S1600536808007204

**Published:** 2008-03-29

**Authors:** Zhichen Zhu, Jin Cui, Mingjie Zhang

**Affiliations:** a Department of Materials Science and Engineering, Tianjin Institute of Urban Construction, Tianjin 300384, People’s Republic of China; b Department of Chemistry, Tianjin University, Tianjin 300072, People’s Republic of China

## Abstract

In the mol­ecule of the title compound, C_28_H_40_N_2_O_2_, the *tert*-butyl group is disordered over two positions; site-occupation factors were kept fixed at 0.5. The morpholine ring has a chair conformation. Intra­molecular O—H⋯N hydrogen bonding results in the formation of a planar six-membered ring, which is oriented at a dihedral angle of 0.70 (3)° with respect to the adjacent aromatic ring. The dihedral angle between the benzene rings is 67.66 (3)°.

## Related literature

For general background, see: Younkin *et al.* (2000 ▶); Gibson & Spitzmesser (2003 ▶). For ring puckering parameters, see: Cremer & Pople (1975 ▶).
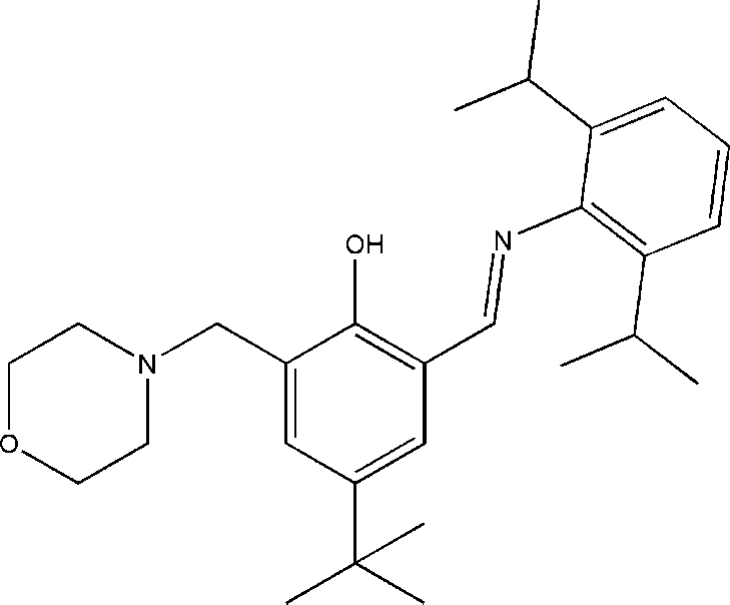


## Experimental

### 

#### Crystal data

C_28_H_40_N_2_O_2_
*M* *_r_* = 436.62
Orthorhombic, 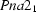

*a* = 10.086 (2) Å
*b* = 20.394 (4) Å
*c* = 12.750 (3) Å
*V* = 2622.8 (10) Å^3^
*Z* = 4
Mo *K*α radiation
μ = 0.07 mm^−1^
*T* = 113 (2) K
0.12 × 0.10 × 0.06 mm

#### Data collection

Rigaku Saturn diffractometer
Absorption correction: multi-scan (Blessing, 1995 ▶) *T*_min_ = 0.992, *T*_max_ = 0.996
25483 measured reflections
4618 independent reflections
4314 reflections with *I* > 2σ(*I*)
*R*_int_ = 0.040

#### Refinement

*R*[*F* ^2^ > 2σ(*F* ^2^)] = 0.037
*wR*(*F* ^2^) = 0.095
*S* = 1.04
4618 reflections
318 parameters
H-atom parameters constrained
Δρ_max_ = 0.18 e Å^−3^
Δρ_min_ = −0.13 e Å^−3^

### 

Data collection: *CrystalClear* (Rigaku/MSC, 2005 ▶); cell refinement: *CrystalClear*; data reduction: *CrystalClear*; program(s) used to solve structure: *SHELXS97* (Sheldrick, 2008 ▶); program(s) used to refine structure: *SHELXL97* (Sheldrick, 2008 ▶); molecular graphics: *SHELXTL* (Sheldrick, 2008 ▶); software used to prepare material for publication: *SHELXTL*.

## Supplementary Material

Crystal structure: contains datablocks global, I. DOI: 10.1107/S1600536808007204/hk2432sup1.cif

Structure factors: contains datablocks I. DOI: 10.1107/S1600536808007204/hk2432Isup2.hkl

Additional supplementary materials:  crystallographic information; 3D view; checkCIF report

## Figures and Tables

**Table 1:** Hydrogen-bond geometry (Å, °)

*D*—H⋯*A*	*D*—H	H⋯*A*	*D*⋯*A*	*D*—H⋯*A*
O2—H2⋯N2	0.82	1.83	2.5630 (18)	148

**Fig. 1.**
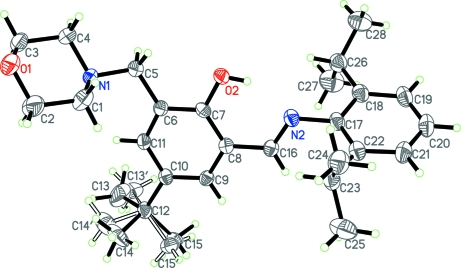
The molecular structure of the title molecule, with the atom-numbering scheme. Displacement ellipsoids are drawn at the 35% probability level.

## supplementary crystallographic information

### Comment

The simple and readily accessible salicylaldiminato ligand family has played an important role in the development of transition metal coordination chemistry, and recently they have been shown to support highly active polymerization centers for both the early and late transition metals (Younkin *et al.*, 2000). Increasing the sizes of the imino and the *o*-phenoxy substituents has a pronounced effect on the activity of the catalyst and the molecular weight of the polymer (Gibson & Spitzmesser, 2003). We have recently prepared the novel title ligand, (I), and report herein its crystal structure

In the molecule of the title compound, (I), (Fig. 1) the bond lengths and angles are within normal ranges. When the crystal structure was solved, the three methyl groups of *tert*-butyl bonded to the phenoxy ring were found to be disordered.

Ring A (O1/N1/C1—C4) is not planar, having total puckering amplitude, Q_T_, of 1.022 (3) Å. It adopts chair [φ = 29.58 (2)° and θ = 56.99 (3)°] conformation (Cremer & Pople, 1975). The intramolecular O—H···N hydrogen bond (Table 1) results in the formation of a planar six-membered ring C (O2/H2/N2/C7/C8/C16), which is oriented with respect to rings B (C6—C11) and D (C17—C22) at dihedral angles of B/C = 0.70 (3)° and C/D = 67.93 (4)°. So, rings B and C are also nearly coplanar. The dihedral angle between rings B and D is B/D = 67.66 (3)°.

### Experimental

2,6-Diisopropylaniline (3.94 g, 22.2 mmol) was added *via* syringe to a solution of 5-*tert*-butyl-2-hydroxy-3-(morpholinomethyl)benzaldehyde (6.15 g, 22.2 mmol) in EtOH (100 ml). The solution was refluxed for 12 h, and then dried over magnesium sulfate, filtered and the volatiles were removed under reduced pressure. Extraction into pentane (10 ml) was followed by cooling to 243 K afforded yellow crystals of (I) (yield; 70%). Spectroscopic analysis: IR (KBr, ν, cm^-1^): 3055.8, 3027.2, 2961.6, 2864.9, 1618.8, 1584.9, 1468.4, 1358.9, 1276.7, 1119.2, 865.7; ^1^H NMR (CDCl_3_, δ, p.p.m.): 1.291 (d, J=6.5 Hz, 12H), 1.355 (s, 9H), 2.619 (s, 4H), 2.933–2.995 (m, 2H), 3.672 (s, 2H), 3.792–3.810 (t, 4H), 7.204(s, 3H), 7.320–7.325 (d, J=2.5 Hz, 1H), 7.539–7.534 (d, J=2.5 Hz, 1H), 8.278 (s, 1H), 9.83(s, 1H)

### Refinement

When the crystal structure was solved, the three methyl groups of *tert*-butyl bonded to the phenoxy ring were found to be disordered. They were each modelled with disorder over two positions with a common carbon atom and occupancies of 50:50. H atoms were positioned geometrically, with O—H = 0.82 Å (for OH) and C—H = 0.93, 0.98, 0.97 and 0.96 Å for aromatic, methine, methylene and methyl H, respectively, and constrained to ride on their parent atoms with *U*_iso_(H) = *xU*_eq_(C,*O*), where *x* = 1.5 for OH and methyl H and *x* = 1.2 for all other H atoms.

### Crystal data

**Table:**

C_28_H_40_N_2_O_2_	*D*_x_ = 1.106 Mg m^−^^3^
*M**_r_* = 436.62	Melting point: 364 K
Orthorhombic, *P**n**a*2_1_	Mo *K*α radiation λ = 0.71073 Å
Hall symbol: P 2c -2n	Cell parameters from 5337 reflections
*a* = 10.086 (2) Å	θ = 1.9–29.7º
*b* = 20.394 (4) Å	µ = 0.07 mm^−^^1^
*c* = 12.750 (3) Å	*T* = 113 (2) K
*V* = 2622.8 (10) Å^3^	Block, yellow
*Z* = 4	0.12 × 0.10 × 0.06 mm
*F*_000_ = 952	

### Data collection

**Table:**

Rigaku Saturn diffractometer	4314 reflections with *I* > 2σ(*I*)
Monochromator: confocal	*R*_int_ = 0.040
*T* = 294(2) K	θ_max_ = 25.0º
ω scans	θ_min_ = 2.3º
Absorption correction: multi-scan(Blessing, 1995)	*h* = −12→11
*T*_min_ = 0.992, *T*_max_ = 0.996	*k* = −24→24
25483 measured reflections	*l* = −15→15
4618 independent reflections	

### Refinement

**Table:**

Refinement on *F*^2^	Hydrogen site location: inferred from neighbouring sites
Least-squares matrix: full	H-atom parameters constrained
*R*[*F*^2^ > 2σ(*F*^2^)] = 0.037	*w* = 1/[σ^2^(*F*_o_^2^) + (0.0588*P*)^2^ + 0.2207*P*] where *P* = (*F*_o_^2^ + 2*F*_c_^2^)/3
*wR*(*F*^2^) = 0.095	(Δ/σ)_max_ = 0.002
*S* = 1.04	Δρ_max_ = 0.18 e Å^−^^3^
4618 reflections	Δρ_min_ = −0.13 e Å^−^^3^
318 parameters	Extinction correction: SHELXL97 (Sheldrick, 2008), Fc^*^=kFc[1+0.001xFc^2^λ^3^/sin(2θ)]^-1/4^
Primary atom site location: structure-invariant direct methods	Extinction coefficient: 0.0157 (15)
Secondary atom site location: difference Fourier map	

### Special details

**Table:**

Geometry. All e.s.d.'s (except the e.s.d. in the dihedral angle between two l.s. planes) are estimated using the full covariance matrix. The cell e.s.d.'s are taken into account individually in the estimation of e.s.d.'s in distances, angles and torsion angles; correlations between e.s.d.'s in cell parameters are only used when they are defined by crystal symmetry. An approximate (isotropic) treatment of cell e.s.d.'s is used for estimating e.s.d.'s involving l.s. planes.
Refinement. Refinement of *F*^2^ against ALL reflections. The weighted *R*-factor *wR* and goodness of fit *S* are based on *F*^2^, conventional *R*-factors *R* are based on *F*, with *F* set to zero for negative *F*^2^. The threshold expression of *F*^2^ > σ(*F*^2^) is used only for calculating *R*-factors(gt) *etc*. and is not relevant to the choice of reflections for refinement. *R*-factors based on *F*^2^ are statistically about twice as large as those based on *F*, and *R*- factors based on ALL data will be even larger.

### Fractional atomic coordinates and isotropic or equivalent isotropic displacement parameters (Å^2^)

**Table:**

	*x*	*y*	*z*	*U*_iso_*/*U*_eq_	Occ. (<1)
O1	0.66312 (13)	0.02738 (7)	0.58337 (11)	0.0474 (4)	
O2	0.44036 (11)	0.16255 (6)	1.01270 (9)	0.0279 (3)	
H2	0.4303	0.1919	1.0559	0.042*	
N1	0.47500 (13)	0.04647 (6)	0.74844 (11)	0.0265 (3)	
N2	0.32848 (13)	0.25917 (7)	1.10594 (11)	0.0280 (3)	
C1	0.53145 (19)	0.10072 (9)	0.68955 (16)	0.0403 (5)	
H1A	0.6101	0.1168	0.7251	0.048*	
H1B	0.4678	0.1363	0.6857	0.048*	
C2	0.5673 (2)	0.07857 (11)	0.58034 (18)	0.0527 (6)	
H2A	0.4883	0.0632	0.5447	0.063*	
H2B	0.6025	0.1154	0.5410	0.063*	
C3	0.6091 (2)	−0.02659 (9)	0.63968 (16)	0.0406 (5)	
H3A	0.6740	−0.0616	0.6427	0.049*	
H3B	0.5317	−0.0430	0.6028	0.049*	
C4	0.57015 (19)	−0.00743 (9)	0.74948 (15)	0.0355 (4)	
H4A	0.5314	−0.0449	0.7848	0.043*	
H4B	0.6485	0.0057	0.7882	0.043*	
C5	0.44409 (19)	0.06580 (9)	0.85631 (14)	0.0352 (4)	
H5A	0.5235	0.0834	0.8887	0.042*	
H5B	0.4181	0.0271	0.8956	0.042*	
C6	0.33465 (16)	0.11632 (8)	0.86387 (13)	0.0262 (4)	
C7	0.33721 (15)	0.16278 (8)	0.94452 (12)	0.0234 (3)	
C8	0.23469 (15)	0.20886 (8)	0.95331 (13)	0.0234 (3)	
C9	0.12971 (16)	0.20779 (7)	0.88116 (13)	0.0247 (3)	
H9A	0.0617	0.2383	0.8879	0.030*	
C10	0.12458 (15)	0.16266 (7)	0.80036 (13)	0.0254 (4)	
C11	0.22915 (16)	0.11738 (8)	0.79479 (13)	0.0260 (4)	
H11A	0.2273	0.0863	0.7415	0.031*	
C12	0.01307 (17)	0.16019 (8)	0.71896 (14)	0.0307 (4)	
C13	−0.0397 (15)	0.0916 (5)	0.6974 (9)	0.071 (3)	0.50
H13A	−0.0129	0.0779	0.6285	0.106*	0.50
H13B	−0.1348	0.0919	0.7015	0.106*	0.50
H13C	−0.0049	0.0617	0.7485	0.106*	0.50
C14	0.0691 (10)	0.1924 (4)	0.6171 (6)	0.061 (2)	0.50
H14A	0.1041	0.2350	0.6334	0.092*	0.50
H14B	−0.0006	0.1966	0.5663	0.092*	0.50
H14C	0.1384	0.1654	0.5889	0.092*	0.50
C15	−0.1030 (10)	0.2032 (6)	0.7539 (9)	0.0307 (19)	0.50
H15A	−0.0786	0.2486	0.7479	0.046*	0.50
H15B	−0.1247	0.1935	0.8256	0.046*	0.50
H15C	−0.1785	0.1946	0.7102	0.046*	0.50
C13'	−0.0535 (11)	0.0920 (4)	0.7330 (8)	0.049 (2)	0.50
H13D	−0.0796	0.0865	0.8049	0.074*	0.50
H13E	0.0082	0.0582	0.7142	0.074*	0.50
H13F	−0.1303	0.0892	0.6888	0.074*	0.50
C14'	0.0708 (11)	0.1619 (4)	0.6092 (6)	0.063 (2)	0.50
H14D	0.1136	0.2033	0.5977	0.095*	0.50
H14E	0.0010	0.1562	0.5588	0.095*	0.50
H14F	0.1344	0.1271	0.6015	0.095*	0.50
C15'	−0.0942 (12)	0.2112 (6)	0.7301 (10)	0.046 (3)	0.50
H15D	−0.0563	0.2541	0.7220	0.069*	0.50
H15E	−0.1340	0.2077	0.7982	0.069*	0.50
H15F	−0.1605	0.2044	0.6772	0.069*	0.50
C16	0.23491 (16)	0.25686 (8)	1.03797 (13)	0.0264 (4)	
H16A	0.1653	0.2866	1.0428	0.032*	
C17	0.32130 (16)	0.30595 (9)	1.18948 (13)	0.0288 (4)	
C18	0.30559 (17)	0.28018 (9)	1.29173 (15)	0.0327 (4)	
C19	0.29501 (18)	0.32502 (10)	1.37352 (15)	0.0379 (4)	
H19A	0.2815	0.3096	1.4413	0.045*	
C20	0.3040 (2)	0.39149 (10)	1.35681 (15)	0.0409 (5)	
H20A	0.2946	0.4205	1.4126	0.049*	
C21	0.3271 (2)	0.41500 (9)	1.25706 (16)	0.0400 (5)	
H21A	0.3356	0.4600	1.2470	0.048*	
C22	0.33791 (17)	0.37310 (9)	1.17069 (15)	0.0331 (4)	
C23	0.3753 (2)	0.40061 (9)	1.06383 (15)	0.0389 (5)	
H23A	0.3678	0.3650	1.0126	0.047*	
C24	0.5192 (2)	0.42388 (10)	1.06373 (17)	0.0443 (5)	
H24A	0.5760	0.3885	1.0851	0.066*	
H24B	0.5288	0.4599	1.1116	0.066*	
H24C	0.5433	0.4378	0.9944	0.066*	
C25	0.2825 (2)	0.45530 (12)	1.0295 (2)	0.0597 (6)	
H25A	0.1927	0.4397	1.0304	0.090*	
H25B	0.3052	0.4690	0.9597	0.090*	
H25C	0.2910	0.4918	1.0766	0.090*	
C26	0.29776 (18)	0.20684 (9)	1.30904 (15)	0.0373 (4)	
H26A	0.3423	0.1858	1.2496	0.045*	
C27	0.1547 (2)	0.18351 (11)	1.3088 (2)	0.0601 (6)	
H27A	0.1120	0.1978	1.2455	0.090*	
H27B	0.1092	0.2015	1.3683	0.090*	
H27C	0.1527	0.1365	1.3123	0.090*	
C28	0.3684 (2)	0.18417 (11)	1.40882 (18)	0.0525 (6)	
H28A	0.4588	0.1988	1.4075	0.079*	
H28B	0.3662	0.1372	1.4126	0.079*	
H28C	0.3245	0.2023	1.4690	0.079*	

### Atomic displacement parameters (Å^2^)

**Table:**

	*U*^11^	*U*^22^	*U*^33^	*U*^12^	*U*^13^	*U*^23^
O1	0.0416 (8)	0.0574 (9)	0.0433 (8)	0.0162 (7)	0.0116 (7)	−0.0014 (7)
O2	0.0244 (6)	0.0340 (7)	0.0254 (6)	0.0025 (5)	−0.0045 (5)	−0.0040 (5)
N1	0.0278 (7)	0.0262 (7)	0.0254 (7)	0.0053 (6)	0.0026 (6)	−0.0005 (6)
N2	0.0264 (7)	0.0319 (7)	0.0259 (7)	−0.0017 (6)	−0.0010 (6)	−0.0038 (6)
C1	0.0386 (10)	0.0301 (9)	0.0523 (13)	0.0022 (8)	0.0144 (10)	0.0011 (8)
C2	0.0518 (12)	0.0587 (13)	0.0476 (13)	0.0186 (10)	0.0214 (11)	0.0139 (11)
C3	0.0388 (10)	0.0409 (10)	0.0421 (11)	0.0123 (8)	−0.0039 (9)	−0.0128 (9)
C4	0.0370 (9)	0.0323 (9)	0.0373 (10)	0.0112 (8)	−0.0018 (8)	−0.0023 (8)
C5	0.0361 (10)	0.0428 (10)	0.0267 (9)	0.0127 (8)	−0.0016 (8)	−0.0039 (8)
C6	0.0252 (8)	0.0300 (8)	0.0233 (8)	0.0032 (6)	0.0026 (7)	−0.0004 (7)
C7	0.0224 (8)	0.0275 (8)	0.0204 (8)	−0.0040 (6)	−0.0003 (7)	0.0030 (6)
C8	0.0227 (8)	0.0241 (8)	0.0235 (8)	−0.0035 (6)	0.0021 (7)	0.0003 (6)
C9	0.0218 (8)	0.0250 (8)	0.0271 (9)	0.0006 (6)	0.0011 (7)	−0.0020 (7)
C10	0.0236 (8)	0.0291 (8)	0.0234 (8)	−0.0026 (6)	0.0020 (7)	−0.0013 (7)
C11	0.0296 (9)	0.0257 (8)	0.0227 (8)	−0.0010 (6)	0.0002 (7)	−0.0034 (7)
C12	0.0269 (8)	0.0373 (9)	0.0281 (9)	0.0023 (7)	−0.0035 (7)	−0.0084 (7)
C13	0.069 (5)	0.051 (4)	0.092 (7)	0.011 (3)	−0.032 (5)	−0.040 (4)
C14	0.042 (3)	0.118 (6)	0.024 (3)	0.011 (4)	−0.008 (2)	0.005 (3)
C15	0.031 (3)	0.035 (4)	0.026 (4)	0.005 (2)	−0.017 (2)	−0.001 (2)
C13'	0.041 (4)	0.034 (3)	0.072 (5)	0.001 (2)	−0.045 (4)	−0.017 (3)
C14'	0.044 (3)	0.109 (6)	0.036 (3)	0.009 (4)	−0.005 (2)	0.002 (4)
C15'	0.057 (4)	0.035 (4)	0.045 (6)	0.014 (3)	−0.017 (3)	−0.006 (4)
C16	0.0256 (8)	0.0252 (8)	0.0285 (9)	−0.0029 (6)	0.0005 (7)	−0.0022 (7)
C17	0.0242 (8)	0.0360 (9)	0.0261 (9)	−0.0023 (7)	−0.0027 (7)	−0.0071 (7)
C18	0.0262 (9)	0.0412 (10)	0.0306 (10)	−0.0007 (7)	−0.0020 (8)	−0.0046 (8)
C19	0.0355 (9)	0.0514 (11)	0.0267 (9)	−0.0003 (8)	0.0009 (8)	−0.0082 (9)
C20	0.0445 (11)	0.0454 (11)	0.0330 (11)	−0.0009 (9)	0.0032 (9)	−0.0143 (9)
C21	0.0484 (11)	0.0324 (9)	0.0392 (11)	−0.0010 (8)	−0.0012 (9)	−0.0095 (8)
C22	0.0349 (9)	0.0350 (9)	0.0295 (9)	−0.0026 (7)	−0.0029 (8)	−0.0047 (7)
C23	0.0489 (11)	0.0355 (10)	0.0323 (10)	−0.0052 (8)	−0.0032 (9)	−0.0072 (8)
C24	0.0429 (11)	0.0461 (11)	0.0440 (11)	0.0007 (9)	0.0053 (10)	−0.0051 (9)
C25	0.0538 (13)	0.0767 (16)	0.0485 (13)	0.0090 (12)	−0.0107 (11)	0.0142 (12)
C26	0.0377 (10)	0.0422 (10)	0.0319 (10)	0.0010 (8)	0.0023 (9)	−0.0007 (9)
C27	0.0497 (13)	0.0569 (13)	0.0738 (17)	−0.0160 (11)	−0.0136 (13)	0.0043 (13)
C28	0.0514 (12)	0.0545 (13)	0.0516 (13)	−0.0024 (10)	−0.0053 (11)	0.0130 (11)

### Geometric parameters (Å, °)

**Table:**

O1—C3	1.423 (3)	C14—H14C	0.9600
O1—C2	1.423 (2)	C15—H15A	0.9600
O2—C7	1.3558 (19)	C15—H15B	0.9600
O2—H2	0.8200	C15—H15C	0.9600
N1—C1	1.453 (2)	C13'—H13D	0.9600
N1—C4	1.459 (2)	C13'—H13E	0.9600
N1—C5	1.464 (2)	C13'—H13F	0.9600
N2—C16	1.282 (2)	C14'—H14D	0.9600
N2—C17	1.432 (2)	C14'—H14E	0.9600
C1—C2	1.508 (3)	C14'—H14F	0.9600
C1—H1A	0.9700	C15'—H15D	0.9600
C1—H1B	0.9700	C15'—H15E	0.9600
C2—H2A	0.9700	C15'—H15F	0.9600
C2—H2B	0.9700	C16—H16A	0.9300
C3—C4	1.506 (3)	C17—C22	1.400 (3)
C3—H3A	0.9700	C17—C18	1.415 (3)
C3—H3B	0.9700	C18—C19	1.391 (3)
C4—H4A	0.9700	C18—C26	1.514 (3)
C4—H4B	0.9700	C19—C20	1.375 (3)
C5—C6	1.513 (2)	C19—H19A	0.9300
C5—H5A	0.9700	C20—C21	1.379 (3)
C5—H5B	0.9700	C20—H20A	0.9300
C6—C11	1.382 (2)	C21—C22	1.398 (3)
C6—C7	1.398 (2)	C21—H21A	0.9300
C7—C8	1.402 (2)	C22—C23	1.521 (3)
C8—C9	1.403 (2)	C23—C25	1.521 (3)
C8—C16	1.457 (2)	C23—C24	1.527 (3)
C9—C10	1.382 (2)	C23—H23A	0.9800
C9—H9A	0.9300	C24—H24A	0.9600
C10—C11	1.404 (2)	C24—H24B	0.9600
C10—C12	1.531 (2)	C24—H24C	0.9600
C11—H11A	0.9300	C25—H25A	0.9600
C12—C15'	1.508 (8)	C25—H25B	0.9600
C12—C14'	1.517 (7)	C25—H25C	0.9600
C12—C13	1.522 (8)	C26—C27	1.519 (3)
C12—C15	1.530 (7)	C26—C28	1.530 (3)
C12—C13'	1.554 (7)	C26—H26A	0.9800
C12—C14	1.561 (7)	C27—H27A	0.9600
C13—H13A	0.9600	C27—H27B	0.9600
C13—H13B	0.9600	C27—H27C	0.9600
C13—H13C	0.9600	C28—H28A	0.9600
C14—H14A	0.9600	C28—H28B	0.9600
C14—H14B	0.9600	C28—H28C	0.9600
			
C3—O1—C2	108.71 (14)	H14B—C14—H14C	109.5
C7—O2—H2	109.5	C12—C15—H15A	109.5
C1—N1—C4	108.69 (14)	C12—C15—H15B	109.5
C1—N1—C5	111.33 (14)	H15A—C15—H15B	109.5
C4—N1—C5	109.53 (13)	C12—C15—H15C	109.5
C16—N2—C17	119.34 (13)	H15A—C15—H15C	109.5
N1—C1—C2	110.06 (16)	H15B—C15—H15C	109.5
N1—C1—H1A	109.6	C12—C13'—H13D	109.5
C2—C1—H1A	109.6	C12—C13'—H13E	109.5
N1—C1—H1B	109.6	H13D—C13'—H13E	109.5
C2—C1—H1B	109.6	C12—C13'—H13F	109.5
H1A—C1—H1B	108.2	H13D—C13'—H13F	109.5
O1—C2—C1	110.94 (17)	H13E—C13'—H13F	109.5
O1—C2—H2A	109.5	C12—C14'—H14D	109.5
C1—C2—H2A	109.5	C12—C14'—H14E	109.5
O1—C2—H2B	109.5	H14D—C14'—H14E	109.5
C1—C2—H2B	109.5	C12—C14'—H14F	109.5
H2A—C2—H2B	108.0	H14D—C14'—H14F	109.5
O1—C3—C4	111.61 (15)	H14E—C14'—H14F	109.5
O1—C3—H3A	109.3	C12—C15'—H15D	109.5
C4—C3—H3A	109.3	C12—C15'—H15E	109.5
O1—C3—H3B	109.3	H15D—C15'—H15E	109.5
C4—C3—H3B	109.3	C12—C15'—H15F	109.5
H3A—C3—H3B	108.0	H15D—C15'—H15F	109.5
N1—C4—C3	111.01 (15)	H15E—C15'—H15F	109.5
N1—C4—H4A	109.4	N2—C16—C8	121.77 (14)
C3—C4—H4A	109.4	N2—C16—H16A	119.1
N1—C4—H4B	109.4	C8—C16—H16A	119.1
C3—C4—H4B	109.4	C22—C17—C18	122.30 (15)
H4A—C4—H4B	108.0	C22—C17—N2	121.22 (15)
N1—C5—C6	113.49 (14)	C18—C17—N2	116.34 (15)
N1—C5—H5A	108.9	C19—C18—C17	117.09 (16)
C6—C5—H5A	108.9	C19—C18—C26	122.42 (17)
N1—C5—H5B	108.9	C17—C18—C26	120.48 (15)
C6—C5—H5B	108.9	C20—C19—C18	121.78 (18)
H5A—C5—H5B	107.7	C20—C19—H19A	119.1
C11—C6—C7	118.20 (14)	C18—C19—H19A	119.1
C11—C6—C5	122.14 (15)	C19—C20—C21	119.79 (17)
C7—C6—C5	119.65 (14)	C19—C20—H20A	120.1
O2—C7—C6	118.90 (14)	C21—C20—H20A	120.1
O2—C7—C8	121.14 (14)	C20—C21—C22	121.81 (18)
C6—C7—C8	119.95 (14)	C20—C21—H21A	119.1
C7—C8—C9	119.60 (14)	C22—C21—H21A	119.1
C7—C8—C16	120.56 (14)	C21—C22—C17	116.98 (18)
C9—C8—C16	119.83 (14)	C21—C22—C23	119.96 (16)
C10—C9—C8	121.83 (14)	C17—C22—C23	122.93 (16)
C10—C9—H9A	119.1	C25—C23—C22	112.08 (18)
C8—C9—H9A	119.1	C25—C23—C24	110.91 (17)
C9—C10—C11	116.59 (14)	C22—C23—C24	110.57 (16)
C9—C10—C12	123.67 (14)	C25—C23—H23A	107.7
C11—C10—C12	119.74 (14)	C22—C23—H23A	107.7
C6—C11—C10	123.83 (15)	C24—C23—H23A	107.7
C6—C11—H11A	118.1	C23—C24—H24A	109.5
C10—C11—H11A	118.1	C23—C24—H24B	109.5
C15'—C12—C14'	110.3 (6)	H24A—C24—H24B	109.5
C15'—C12—C13	113.6 (9)	C23—C24—H24C	109.5
C14'—C12—C13	89.3 (6)	H24A—C24—H24C	109.5
C14'—C12—C15	123.4 (6)	H24B—C24—H24C	109.5
C13—C12—C15	108.2 (8)	C23—C25—H25A	109.5
C15'—C12—C10	116.1 (6)	C23—C25—H25B	109.5
C14'—C12—C10	110.0 (5)	H25A—C25—H25B	109.5
C13—C12—C10	114.2 (6)	C23—C25—H25C	109.5
C15—C12—C10	110.2 (5)	H25A—C25—H25C	109.5
C15'—C12—C13'	107.2 (7)	H25B—C25—H25C	109.5
C14'—C12—C13'	107.0 (6)	C18—C26—C27	111.01 (17)
C15—C12—C13'	98.6 (7)	C18—C26—C28	113.29 (16)
C10—C12—C13'	105.6 (4)	C27—C26—C28	110.46 (18)
C15'—C12—C14	92.7 (6)	C18—C26—H26A	107.3
C13—C12—C14	111.3 (6)	C27—C26—H26A	107.3
C15—C12—C14	106.2 (6)	C28—C26—H26A	107.3
C10—C12—C14	106.5 (4)	C26—C27—H27A	109.5
C13'—C12—C14	129.0 (5)	C26—C27—H27B	109.5
C12—C13—H13A	109.5	H27A—C27—H27B	109.5
C12—C13—H13B	109.5	C26—C27—H27C	109.5
H13A—C13—H13B	109.5	H27A—C27—H27C	109.5
C12—C13—H13C	109.5	H27B—C27—H27C	109.5
H13A—C13—H13C	109.5	C26—C28—H28A	109.5
H13B—C13—H13C	109.5	C26—C28—H28B	109.5
C12—C14—H14A	109.5	H28A—C28—H28B	109.5
C12—C14—H14B	109.5	C26—C28—H28C	109.5
H14A—C14—H14B	109.5	H28A—C28—H28C	109.5
C12—C14—H14C	109.5	H28B—C28—H28C	109.5
H14A—C14—H14C	109.5		
			
C4—N1—C1—C2	57.0 (2)	C9—C10—C12—C15	−12.5 (6)
C5—N1—C1—C2	177.71 (15)	C11—C10—C12—C15	167.6 (5)
C3—O1—C2—C1	60.1 (2)	C9—C10—C12—C13'	−118.0 (5)
N1—C1—C2—O1	−60.7 (2)	C11—C10—C12—C13'	62.1 (5)
C2—O1—C3—C4	−58.5 (2)	C9—C10—C12—C14	102.2 (4)
C1—N1—C4—C3	−55.6 (2)	C11—C10—C12—C14	−77.6 (4)
C5—N1—C4—C3	−177.41 (16)	C17—N2—C16—C8	−177.94 (15)
O1—C3—C4—N1	57.4 (2)	C7—C8—C16—N2	0.8 (2)
C1—N1—C5—C6	66.21 (19)	C9—C8—C16—N2	179.50 (15)
C4—N1—C5—C6	−173.56 (15)	C16—N2—C17—C22	−70.8 (2)
N1—C5—C6—C11	34.2 (2)	C16—N2—C17—C18	113.33 (18)
N1—C5—C6—C7	−147.46 (15)	C22—C17—C18—C19	5.7 (3)
C11—C6—C7—O2	−179.73 (14)	N2—C17—C18—C19	−178.39 (15)
C5—C6—C7—O2	1.8 (2)	C22—C17—C18—C26	−175.61 (16)
C11—C6—C7—C8	−0.2 (2)	N2—C17—C18—C26	0.3 (2)
C5—C6—C7—C8	−178.66 (15)	C17—C18—C19—C20	−2.2 (3)
O2—C7—C8—C9	179.79 (14)	C26—C18—C19—C20	179.14 (18)
C6—C7—C8—C9	0.3 (2)	C18—C19—C20—C21	−1.5 (3)
O2—C7—C8—C16	−1.5 (2)	C19—C20—C21—C22	1.9 (3)
C6—C7—C8—C16	178.97 (14)	C20—C21—C22—C17	1.4 (3)
C7—C8—C9—C10	−0.6 (2)	C20—C21—C22—C23	−174.63 (19)
C16—C8—C9—C10	−179.24 (15)	C18—C17—C22—C21	−5.3 (3)
C8—C9—C10—C11	0.7 (2)	N2—C17—C22—C21	179.01 (15)
C8—C9—C10—C12	−179.16 (15)	C18—C17—C22—C23	170.59 (17)
C7—C6—C11—C10	0.4 (2)	N2—C17—C22—C23	−5.1 (3)
C5—C6—C11—C10	178.80 (16)	C21—C22—C23—C25	−55.3 (2)
C9—C10—C11—C6	−0.6 (2)	C17—C22—C23—C25	128.9 (2)
C12—C10—C11—C6	179.22 (15)	C21—C22—C23—C24	69.0 (2)
C9—C10—C12—C15'	0.6 (6)	C17—C22—C23—C24	−106.78 (19)
C11—C10—C12—C15'	−179.2 (6)	C19—C18—C26—C27	85.8 (2)
C9—C10—C12—C14'	126.8 (4)	C17—C18—C26—C27	−92.8 (2)
C11—C10—C12—C14'	−53.0 (4)	C19—C18—C26—C28	−39.2 (3)
C9—C10—C12—C13	−134.6 (6)	C17—C18—C26—C28	142.27 (18)
C11—C10—C12—C13	45.6 (6)		

### Hydrogen-bond geometry (Å, °)

**Table:**

*D*—H···*A*	*D*—H	H···*A*	*D*···*A*	*D*—H···*A*
O2—H2···N2	0.82	1.83	2.5630 (18)	148
